# Blood Glucose Levels May Aid the Decision for CT Scan in Minor Head Trauma

**DOI:** 10.1155/2019/1065254

**Published:** 2019-04-09

**Authors:** George A. Alexiou, Athanasios Sotiropoulos, Georgios D. Lianos, Andreas Zigouris, Dimitrios Metaxas, Anastasios Nasios, Evaggelos Michos, Michail Mitsis, Dimitrios Pachatouridis, Spyridon Voulgaris

**Affiliations:** ^1^Department of Neurosurgery, University Hospital of Ioannina, Ioannina, Greece; ^2^Department of Surgery, University Hospital of Ioannina, Ioannina, Greece

## Abstract

Traumatic brain injury has been associated with increased blood glucose levels. In the present study, we set out to investigate if blood glucose level in mild head trauma could predict the need for CT. One hundred fifty-nine patients with minor TBI (GCS 13-15) and a mean age of 44.8 ± 23.8 years were included in the study. The most common mechanism of trauma was falls. Patients with positive CT findings had significantly higher glucose levels than patients with negative CT findings. Using ROC curve analysis, serum glucose levels higher than 120 mg dl^−1^ were the optimal cutoff value for the detection of patients with positive CT findings with a sensitivity of 74.4% and a specificity of 90.7%. Serum glucose level evaluation at presentation in the emergency department may aid CT decision-making in mild TBI.

## 1. Introduction

Traumatic brain injury (TBI) is the leading cause of death and disability in persons under 40 years of age [1]. Although CT is indicated for moderate and severe head trauma, there is a debate over the indications and yield of neuroimaging for minor head trauma. Especially for pediatric head trauma patients, the risk from ionizing radiation is considerable and carries a risk of leukemia and brain tumor, especially in children under 10 years of age [2].

TBI has been associated with increased blood glucose levels. Hyperglycemia has been associated with poor outcome and increased mortality [3]. There are several mechanisms by which hyperglycemia is induced after TBI [4]. We have previously reported that patients with severe TBI had significantly higher serum glucose levels compared to patients with mild TBI. Patients with a serum glucose level greater than 151 mg dl^−1^ will probably develop coagulopathy, which is associated with increased morbidity and mortality [5]. In the present study, we set out to investigate if blood glucose levels at presentation of patients with mild head trauma could predict the need for CT.

## 2. Material and Methods

In this retrospective study, we included patients with blunt traumatic head trauma, aged ≥18 years, with a GCS score ≥ 13, with serum glucose levels available at presentation in the emergency department, who underwent noncontrast brain CT scan, and admitted in the neurosurgical or surgical department during a four-year period. The mechanism of injury, admission blood glucose levels, presenting symptoms, presence of other injuries, presence of scalp hematoma, alcohol intoxication, and head CT findings were recorded. The presence of positive CT findings was defined as lesions related to the trauma and included skull fracture, skull base fracture, depressed skull fracture, epidural/subdural hematoma, posttraumatic subarachnoid hemorrhage, and contusion. We excluded patients with diabetes mellitus, penetrating TBI, multisystem trauma, and age < 18 years. The study was conducted in accordance with the World Medical Association Declaration of Helsinki.

## 3. Statistical Analysis

Continuous data were expressed as mean ± standard deviation. To compare glucose levels between patients with positive and negative CT findings, we used the two-sided, nonparametric Mann–Whitney *U*-test. Receiver operating characteristic (ROC) analysis was used to define the value more efficiently detecting patients with positive CT findings. A 2-sided *p* value < 0.05 was considered statistically significant.

## 4. Results

One hundred fifty-nine patients were included in the study (Table 1). The mean age of the patients was 44.8 ± 23.8 years (range: 18 to 95) and 69.2% were male. The most common mechanism of trauma was falls (47.2%) followed by motor vehicle accidents (35.8%). Forty-two (26.4%) patients had a negative CT. Detailed positive CT findings are described in Table 2. The mean glucose levels in the emergency department at presentation were 126.3 ± 27.8 mg dl^−1^. At presentation, 10 patients had a GCS of 13, 23 patients had a GCS of 14, and the remaining 126 patients had a GCS of 15. Patients with positive CT findings had significantly higher glucose levels than patients with negative CT findings (135.7 ± 26.5 vs. 102.4 ± 12.4, respectively, *p* < 0.0001). Using the ROC curve, it was found that a serum glucose of 120 mg dl^−1^ was the threshold for the detection of patients with positive CT findings with a sensitivity of 74.4% and a specificity of 90.7%. All patients with blood glucose levels over 128 mg dl^−1^ had positive CT findings. A GCS score lower or equal to 14 had a 27.2% sensitivity and 95.6% specificity for the detection of patients with positive CT findings. No significant difference was found in glucose levels between patients with and without alcohol intoxication (*p* = 0.8). Patients with loss of consciousness and posttraumatic amnesia more frequently had a positive CT, but the difference was not statistically significant (*p* = 0.17 and *p* = 0.1, respectively).

## 5. Discussion

The present study showed that in patients with mild head trauma blood, glucose levels at presentation were significantly higher in patients with positive CT findings than in patients with a negative CT. A cutoff value of 120 mg dl^−1^ could detect patients with a need for CT with 74.4% sensitivity and 90.7% specificity. To the best of our knowledge, no previous study investigated the value of blood glucose levels at presentation as a means to predict the need for CT in minor TBI.

Traumatic brain injury is a serious health issue. In the US, there were 822 TBI visits per 100 000 person-years in 2010 and the majority were classified as minor and discharged. An increase in incidence has been reported mainly for concussion or unspecified head injury [6]. Apart from radiation exposure, ordering a CT is associated with excessive cost, not to mention the waiting hours in the emergency department, if CT is not readily available, and the false-positive findings or overdiagnosis [7]. Clinical decision rules for adults have also been developed [8]; however, their impact on CT use is limited [9].

To date, several blood biomarkers have been studied over the past years as potential candidates for safely predicting the chances of a brain injury. Bazarian et al. recently studied the value of a biomarker test combining ubiquitin C-terminal hydrolase-L1 (UCH-L1) and glial fibrillary acidic protein (GFAP) levels to predict traumatic intracranial injuries on head CT. This prospective, multicenter, observational trial recruited 1959 patients with GCS 9-15. Among these patients, only 8 were in need of neurosurgical intervention and 125 had positive head CT. Overall, the test sensitivity was found to be 97.6% and the negative predictive value (NPV) was 99.6%. For lesions with a need for neurosurgical intervention, the test had 100% sensitivity and 100% NPV [10]. The value of S100B as a serum biomarker has also been investigated in several studies [11, 12]. S100B is a protein mainly found in glial cells and increases rapidly after head trauma [13]. Several studies have demonstrated a 98% sensitivity and 100% sensitivity for clinically important injuries [14, 15]. Calcagnile et al. studied 726 patients with mild TBI (GCS 14-15) and found that measuring S100B may reduce CT usage and is cost effective [12]. Although the previously discussed biomarkers might have higher sensitivity for the detection of patients with positive CT findings than serum glucose levels, these are not readily available in every center and have a certain cost. Blood glucose level assessment is inexpensive and is usually routinely performed in the emergency department. Thus, blood glucose levels may be used to aid the decision for CT scan in minor head trauma.

Increased blood glucose levels can be found after TBI. Stress response has been suggested as a mechanism of hyperglycemia after TBI by the induction of catecholamines that in turn leads to a decrease in insulin secretion [16]. Systemic inflammatory response syndrome (SIRS) has been frequently found after TBI, and hyperglycemia was significantly more frequent in these patients [17]. Pituitary and/or hypothalamic dysfunction has also been proposed as a causative mechanism [4]. Hyperglycemia has been associated with a higher frequency of coagulopathy occurrence and unfavorable prognosis. In a study of 44 children with moderate and severe TBI, increased blood glucose levels were associated with higher mortality, extended duration of mechanical ventilation, and intensive care stay [18]. To our knowledge, the present study is the first to investigate the role of serum glucose levels at presentation in the emergency department for CT decision making in mild TBI.

Our study has several limitations. First, this was a retrospective study. Second, the number of patients with negative CT findings was limited mainly due to the fact that not all patients with a negative CT had serum glucose levels evaluated or were hospitalized. There is certainly a need for a large prospective study to verify our preliminary observations and to investigate the possible correlation between serum glucose levels and type of abnormal CT findings or findings that require neurosurgical intervention. Furthermore, there is a need to clarify if the sensitivity or specificity of serum glucose levels differs according to GCS for the detection of patients with positive CT findings. Finally, it would be also interesting to evaluate the predictive role of blood glucose levels in children with minor head trauma, given the increased risk of exposure to ionizing radiation in this age group.

## Figures and Tables

**Table 1:** Patients' demographics and characteristics.

Age (years)	44.8 ± 23.8
Sex	
Male	110 (69.2%)
Female	49 (30.8%)
GCS score	
13	10 (6.3%)
14	23 (14.5%)
15	126 (79.2%)
Mechanism of injury	
Fall	75 (47.2%)
Motor vehicle accident	57 (35.8%)
Assault	13 (8.2%)
Others	14 (8.8%)
Vomiting	
Yes	57 (35.8)
No	98 (61.6%)
Unknown	4 (2.5%)
Loss of consciousness	
Yes	69 (43.3%)
No	85 (53.5%)
Unknown	5 (3.2%)
Posttraumatic amnesia	
Yes	72 (45.3%)
No	84 (52.8%)
Unknown	3 (1.9%)
Alcohol intoxication	
Yes	27 (17%)
No	132 (83%)

**Table 2:** Positive CT findings.

Acute epidural hematoma	11 (9.4%)
Acute subdural hematoma	31 (26.5%)
Posttraumatic subarachnoid	27 (23%)
Contusion	26 (22.2%)
Skull fracture	29 (24.8%)
Depressed skull fracture	7 (6%)
Skull base fracture	9 (7.7%)

## Data Availability

The data used to support the findings of this study are available from the corresponding author upon request.
